# *Xanthomonas* spp. Infecting Araceae and Araliaceae: Taxonomy, Phylogeny, and Potential Virulence Mechanisms

**DOI:** 10.3390/biology14070766

**Published:** 2025-06-25

**Authors:** Shu-Cheng Chuang, Shefali Dobhal, Lisa M. Keith, Anne M. Alvarez, Mohammad Arif

**Affiliations:** 1 Department of Plant and Environmental Protection Sciences, University of Hawaii at Manoa, Honolulu, HI 96822, USA; chuangsc@hawaii.edu (S.-C.C.); shefali@hawaii.edu (S.D.); alvarez@hawaii.edu (A.M.A.); 2 Tropical Plant Genetic Resources and Disease Research Unit, Agricultural Research Service, USDA, Hilo, HI 96720, USA; lisa.keith@usda.gov

**Keywords:** xanthomonads, *Xanthomonas euvesicatoria*, panax, anthurium, taxonomy, Araceae, Araliaceae

## Abstract

The genus *Xanthomonas* (family Xanthomonadaceae) comprises 39 validly published species and is associated with a broad host range, infecting hundreds of monocot and dicot plants worldwide. While many *Xanthomonas* species are notorious for causing leaf spot and blight diseases in major agricultural crops, less attention has been given to their impact on ornamental plants. In Hawaii and other key production regions, xanthomonads have posed persistent threats to popular ornamentals in the Araceae and Araliaceae families. This review synthesizes the evolving phylogenetic and taxonomic framework of Xanthomonas strains isolated from Araceae and Araliaceae, highlighting recent advances enabled by multilocus sequence analysis and whole genome sequencing. We discuss the reclassification of key pathovars, unresolved phylogenetic placements, and the challenges of pathovar delineation within these plant families. Additionally, we examine current knowledge of molecular determinants of pathogenicity, including gene clusters involved in exopolysaccharide and lipopolysaccharide biosynthesis, flagellar assembly, cell-wall-degrading enzymes, and secretion systems (types II, III, and VI). Comparative genomics and functional studies reveal that significant gaps remain in our understanding of the genetic basis of host adaptation and virulence in these xanthomonads. Addressing these knowledge gaps will be crucial for developing effective diagnostics and management strategies for bacterial diseases in ornamental crops.

## Simple Summary

*Xanthomonas* is a large genus of plant-associated bacteria that cause disease on various crops. But their involvement in ornamental plant diseases, especially in the Araceae and Araliaceae families, has been less studied. This review compiles recent information about their taxonomy, phylogenetic relationships, and potential virulence mechanisms of Xanthomonas strains affecting these ornamental hosts. Using advanced tools, researchers have reclassified many of these strains into distinct species and pathovars and revealed their evolutionary relationships. Despite this progress, the placement of several strains remains unresolved, especially those from lesser-studied genera like *Epipremnum* and *Rhaphidophora*. The review also discusses genes associated with bacterial pathogenicity, such as those involved in secretion systems, exopolysaccharide production, and host cell wall degradation. These molecular insights, combined with future genomic and functional studies, are essential to better define species boundaries and how *Xanthomonas* adapts and causes disease in different plant hosts. This knowledge is key to understanding how these bacteria cause disease in ornamental plants and to guiding future research and control efforts.

## 1. Introduction to *Xanthomonas*

The genus *Xanthomonas* (from Greek: xanthos, “yellow”, and monas, “entity”) comprises Gram-negative, rod-shaped, aerobic, polar monotrichous, and xanthomonadin-producing bacteria. *Xanthomonas* belongs to the family Xanthomonadaceae (syn. Lysobacteraceae), order Xanthomonadales (syn. Lysobacterales), class Gammaproteobacteria, and phylum Pseudomonadota (formerly Proteobacteria) [1,2,3,4]. The genus currently includes 39 validly published species and several others with pending or invalid status [5] (LPSN, accessed 22 June 2025). The type species is *Xanthomonas campestris* [1].

*Xanthomonas* is a major group of phytopathogenic and plant-associated bacteria, collectively infecting more than 400 plant species across both monocots and dicots [6,7,8,9,10,11]. Many species and pathovars cause economically significant diseases worldwide. For example, *X. oryzae* pathovars cause bacterial leaf blight and leaf streak of rice (*Oryza sativa*), *X. campestris* pv. *campestris* causes black rot in crucifers, and *X. citri* pv. *citri* and *X. citri* pv. *malvacearum* cause citrus canker and cotton bacterial blight, respectively. The bacterial leaf spot of tomatoes and peppers is caused by four xanthomonads: *X. euvesicatoria* pv. *euvesicatoria*, *X. euvesicatoria* pv. *perforans*, *X. hortorum* pv. *gardneri*, and *X. vesicatoria*. Other notable diseases include the bacterial spot and canker of stone fruits and almonds (*X. arboricola* pv. *pruni*) and walnut blights (*X. arboricola* pv. *juglandis*) [12,13,14,15].

**Figure 1:**
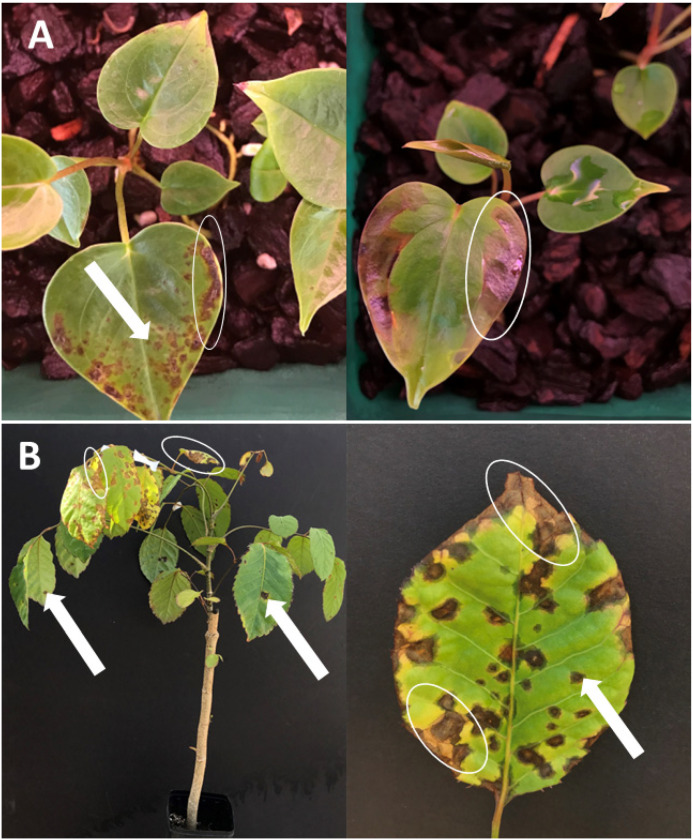
Leaf blight (white circle) and leaf spot (white arrow) symptoms on infected plants in the Araceae and Araliaceae families. (**A**) Water-soaked lesions of leaf spot and leaf blight on the leaves of *Anthurium andraeanum* caused by *X. phaseoli* pv. *dieffenbachiae* (syn. *X. axonopodis* pv. *dieffenbachiae* and *X. campestris* pv. *dieffenbachiae*). (**B**) Water-soaked lesions surrounding of leaf spot and leaf blight symptoms on the leaves of *Polyscias guilfoylei* caused by *X. euvesicatoria* pv. *polysciadis* (formerly *X. hortorum* pv. *hederae*).

In addition to food crops, *Xanthomonas* spp. also cause substantial losses in ornamentals, affecting plants such as anthurium (Araceae), begonia (Begoniaceae), hibiscus (Malvaceae), poinsettia (Euphorbiaceae), pelargonium and geranium (Geraniaceae), English ivy (Araliaceae), and zinnia (Asteraceae) [16,17,18]. This review focuses on bacterial leaf blight and spot diseases caused by *Xanthomonas* spp. on economically important ornamentals in the Araceae and Araliaceae families, particularly those affecting *Anthurium* spp. (Araceae) and *Polyscias* spp. (Araliaceae) in Hawaii, Florida, California, and other major production regions [19,20,21,22,23,24]. Typical leaf blight and spot symptoms on *A. andraeanum* and *P. guilfoylei* are shown in Figure 1.

## 2. Taxonomy, Host Range, and Phylogeny of *Xanthomonas*

*Bacterium hyacinthi* [25], later reclassified as *Xanthomonas hyacinthi* [26], was the first recognized xanthomonad plant pathogen. Early plant-pathogenic bacteria were classified as *Pseudomonas* or *Phytomonas* based on colony pigmentation and pathogenicity [27]. Dowson [1] established the genus *Xanthomonas* for Gram-negative, yellow-pigmented, polar-flagellated bacteria, distinguishing them from *Bacterium* and *Pseudomonas*.

Later, additional xanthomonads were described as pathogens with specific host ranges, highlighting their genetic and phenotypic diversity. Initially, distinguishing *Xanthomonas* species was challenging because biochemical and physiological features alone provided insufficient resolution [28]. As a result, early taxonomy relied on the “new host/new species” concept, where isolates from different hosts were classified as separate species [28,29,30]. This host-based approach led to a proliferation of species, with 60 recognized by Burkholder in 1957 and over 200 reported by 1974. Before the Eighth Edition of Bergey’s Manual [31], it was recognized that many of these species lacked sufficient bacteriological and physiological distinction. Consequently, the taxonomy was revised, consolidating the genus into five species: *X. campestris*, *X. albilineans*, *X. ampelina*, *X. axonopodis*, and *X. fragariae*. Subsequently, *X. ampelina* was transferred to the genus *Xylophilus* as *Xylophilus ampelina* [32]. To retain meaningful distinctions among plant-pathogenic strains, the pathovar system was introduced, allowing differentiation based on host range within the broader species framework [33].

Over time, more genotypic analyses—including GC content determination, DNA fingerprinting, DNA–DNA hybridization (DDH), ribosomal RNA sequencing, and multilocus sequence analysis (MLSA)—were developed and combined with morphological, biochemical, and physiological characteristics to clarify the taxonomy and nomenclature of *Xanthomonas* [26,34,35,36]. Based on DDH characterization, Vauterin et al. [26] proposed reclassifying xanthomonads into 20 species. The classification by DDH corresponded with the grouping results obtained by amplified fragment length polymorphism (AFLP) and repetitive-element palindromic polymerase chain reaction (rep-PCR) [34,35]. In contrast, a phylogenetic analysis of the 16S rRNA gene showed limited taxonomic resolution among the 20 *Xanthomonas* species, grouping 15 species into a large cluster, along with two other clusters (the *X. sacchari* cluster and the *X. albilineans*–*X. translucens*–*X. hyacinthi* cluster). This lack of resolution was attributed to the highly conserved nature of the 16S rRNA sequence within *Xanthomonas* [37].

**Figure 2:**
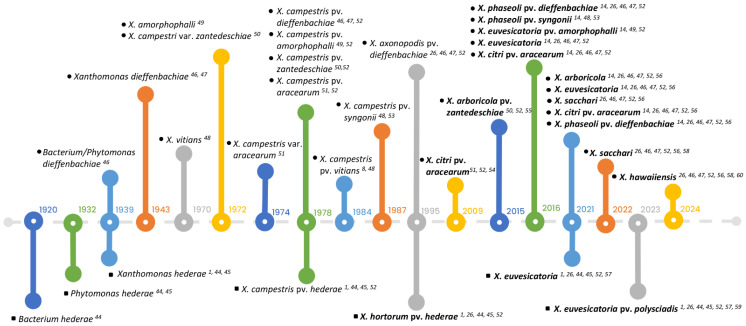
The history of nomenclatural changes of xanthomonads isolated from Araceae and Araliaceae from 1920 to 2023. The upper part of the timeline shows the identification and reclassification of strains isolated from Araceae (indicated by black circles), while the lower part highlights the changes of strains isolated from Araliaceae (represented by black squares). Recent taxonomic names for strains are highlighted in bold. References for each event related to the strains are indicated by superscript numbers and are listed consecutively: [1,8,14,26,44,45,46,47,48,49,50,51,52,53,54,55,56,57,58,59,60].

Although the polyphasic approach described above was implemented to achieve better resolution of *Xanthomonas* spp., debate and controversy have persisted [38,39]. Additional species were described and published using valid and sometimes invalid criteria (https://lpsn.dsmz.de/genus/xanthomonas, accessed on 22 June 2025). In today’s context, the whole genome sequencing technology provides more detailed and comprehensive genomic information, setting a higher standard for species delineation. The current major standards for delineating bacterial species include cut-off values of 70% digital DNA–DNA hybridization (dDDH) and 95% average nucleotide identity (ANI) [40,41,42,43]. Nevertheless, a standard is still lacking for designating subspecific levels based on genomic relatedness, and according to Young et al. [39] and Constantin et al. [14], the resolution of pathovars in phylogenetic trees remains inadequate. The term “pathovar” technically refers only to a group of strains with distinct pathogenicity on a specific host or host range and does not represent a formal taxonomic rank. Such a designation must be supported by data from pathogenicity and host range tests. In this context, we describe the nomenclatural evolution of *Xanthomonas* strains associated with hosts belonging to the Araceae and Araliaceae (Figure 2).

### 2.1. Xanthomonads Associated with the Araceae

The first report of a bacterial leaf blight disease affecting the Araceae was a leaf blight of *Dieffenbachia picta* (syn. *Dieffenbachia seguine*) in New Jersey (USA), originally attributed to *Bacterium dieffenbachiae* (syn. *Phytomonas dieffenbachiae*) [46]. The pathogen was later renamed *Xanthomonas dieffenbachiae* [47]. In 1970, Wehlburg described another xanthomonad isolated from *Syngonium podophyllum* and identified the causal agent of leaf blight as *X. vitians*, a bacterium primarily known for causing leaf spot on lettuce, but which had not yet been reported on *Syngonium*. In 1972 and 1974, three xanthomonads isolated from other genera of Araceae were described: *X. amorphophalli* from *Amorphophallus campanulatus* in India [49], *X. campestris* var. *zantedeschiae* from *Zantedeschia aethiopica* in South Africa [50], and *X. campestris* var. *aracearum* from *Xanthosoma sagittifolium* in Guadeloupe [51]. Furthermore, due to their similar results in bacteriological tests—including physiological and biochemical reactions resembling those of *X. campestris*—it was recommended that these four xanthomonads be renamed at the subspecific level, using the term “pathovar” rather than “variant.” Thus, they were named *X. campestris* pv. *amorphophalli*, *X. campestris* pv. *aracearum*, *X. campestris* pv. *dieffenbachiae*, and *X. campestris* pv. *zantedeschiae* [52].

The host range of the four aforementioned pathovars covers ten species in eight aroid genera, including *Aglaonema*, *Amorphophallus*, *Anthurium*, *Dieffenbachia*, *Philodendron*, *Syngonium*, *Xanthosoma*, and *Zantedeschia*. One *Syngonium* strain was identified as *X. campestris* pv. *vitians*, which mainly causes leaf spot on lettuce [8]. Six other strains isolated from Syngonium podophyllum cultivars ‘Cream’ and ‘White Butterfly’ were distinguished from pathovars *dieffenbachiae*, *zantedeschiae*, and *vitians* on aroids and were proposed as *X. campestris* pv. *syngonii* [53]. Notably, *X. campestris* pv. *dieffenbachiae* was isolated from six araceous species: *Aglaonema*, *Anthurium*, *Dieffenbachia*, and *Philodendron*, as well as from *Dracaena fragrans* in the Asparagaceae family [8,33,61]. Subsequently, the extended host range of *X. campestris* pv. *dieffenbachiae* included *Xanthosoma sagittifolium*, an edible aroid commonly called cocoyam [62], as well as *Colocasia*, *Alocasia*, *Caladium*, *Epipremnum*, *Spathiphyllum*, and *Rhaphidophora* [61]. In contrast, *X. campestris* pv. *zantedeschiae* and *X. campestris* pv. *syngonii* showed host specificity on *Zantedeschia aethiopica* and *Syngonium podophyllum*, respectively [50,53].

**Figure 3:**
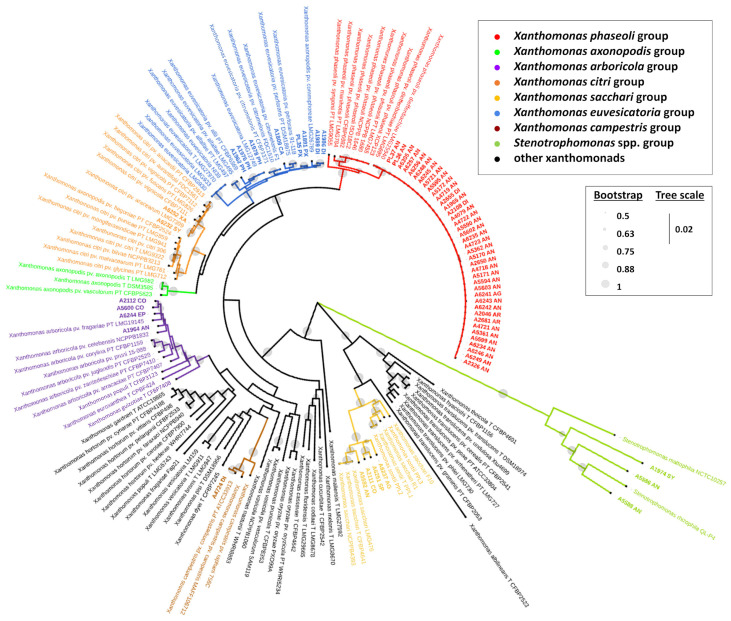
Phylogenetic tree of *Xanthomonas* spp. based on the concatenated nucleotide sequences of the partial regions of five housekeeping genes (*atpD*, *dnaA*, *dnaK*, *gltA*, and *gyrB*). The abbreviations in the strain names are the following: AG = *Aglaonema*; AN = *Anthurium*; AR = Aroid; CA = *Caladium*; CO = *Colocasia*; DI = *Dieffenbachia*; EP = *Epipremnum*; PH = *Philodendron*; PX = *Polyscias guilfoylei* (Panax); SP = *Spathiphyllum*; SY = *Syngonium*; XA = *Xanthosoma*; PT = pathotype strain; and T = type strain. The names of the strains isolated from Araceae and Araliaceae are shown in bold. Reproduced, by permission, from [57,60], *Xanthomonas* strains isolated from hosts in the Araceae reveal diverse phylogenetic relationships and origins. Phytopathology, 114(8):1963-1974. © The American Phytopathological Society.

With the introduction of DDH analyses, the pathovar dieffenbachiae in *X. campestris* was reclassified into *X. axonopodis*, while pathovars *syngonii* and *zantedeschiae* retained their original classification under *X. campestris* [26]. Ah-You et al. [54] proposed that some aroid strains, rather than anthurium strains, should be reclassified under *X. citri* based on DDH, MLSA (*atpD*, *dnaK*, and *gyrB*), and amplified fragment length polymorphism (AFLP) analyses. Further MLSA analyses and whole genome studies were conducted on aroid strains, and the pathovar *zantedeschiae*, known for its host specificity to *Zantedeschia*, was placed in *X. arboricola* [55] whereas pathovar dieffenbachiae infecting *Dieffenbachia*, *Aglaonema*, and primarily *Anthurium*, and pathovar *syngonii* restricted to *Syngonium* were reclassified under *X. phaseoli* [14,56,57,63]; pathovar amorphophalli isolated from Amorphophallus and some strains originally isolated from *Philodendron*, *Dieffenbachia*, and *Caladium* were redesignated as *X. euvesicatoria* [14,56,57]; pathovar aracearum isolated from various Araceae genera was placed under *X. citri*, and four other strains isolated from *Anthurium*, *Aglaonema*, *Colocasia*, and *Spathiphyllum* were grouped with *X. sacchari* [14,54,56,57,58,63]. In the maximum likelihood phylogenetic tree (Figure 3) presented in Chaung et al. [57], over fifty aroid strains were distributed among six *Xanthomonas* species groups (*X. arboricola*, *X. campestris*, *X. citri*, *X. euvesicatoria*, *X. phaseoli*, and *X. sacchari*), aligning closely with groupings reported in previous studies. Meanwhile, three additional aroid strains clustered within the *Stenotrophomonas* spp. group and were subsequently identified as two new species, *S. aracearum* and *S. oahuensis* [57,60]. Furthermore, Chuang et al. [60] identified the spathiphyllum and the colocasia strains, formerly in the *X. sacchari* phylogenetic group, as a novel species, *X. hawaiiensis*, based on ANI and dDDH values lower than the delineation of new species. Notably, some strains of the phylogenetically related species, *X. sacchari*, were considered as commensal bacteria because of the absence of type III secretion system (T3SS) and type III effectors (T3Es) [64,65]. The pathogenicity of *X. hawaiiensis* on Araceae remains uncertain.

### 2.2. Xanthomonads Associated with the Araliaceae

The causal agent of bacterial leaf spot disease on *Hedera helix* L. (English ivy), an araliaceous plant, was first identified as *Bacterium hederae* in France [44] and later renamed *Phytomonas hederae* by Burkholder and Guterman in 1932 [45]. After Dowson [1] designated the genus *Xanthomonas* for monotrichous bacteria producing a yellow xanthomonadin pigment (among other bacteriological characteristics), *P. hederae* was changed to *Xanthomonas hederae*. With the introduction of pathovar designations for *Xanthomonas*, the strains isolated from *H. helix* were designated as the pathovar *X. campestris* pv. *hederae* [52]. DNA homology data and Biolog characteristics supported the grouping of pathovar *hederae* with two other pathovars, *pelargonii* and *vitians*. Consequently, all three pathogens were reclassified into a new species, *X. hortorum*, and named *X. hortorum* pv. *hederae*, *X. hortorum* pv. *pelargonii*, and *X. hortorum* pv. *vitians* [26].

Since 1920, leaf spot diseases have been reported worldwide on various Hedera species and cultivars. These include 12 *H. helix* varieties in the United States [66]; *H. helix* and *H. canariensis* in Japan [67]; *H. helix* in New Zealand, Korea, Greece, Turkey, and Taiwan [68,69,70,71]; *H. hibernica* in Slovenia [72]; and *H. nepalensis* var. *sinensis* in China [73]. The host range of this pathogen has expanded to other members of the Araliaceae family, such as *Heptapleurum actinophyllum* (syn. *Brassaia actinophylla* and *Schefflera actinophylla*; “*Heptapleurum actinophyllum* (Endl.) [74,75], *Fatsia japonica*, *Plerandra elegantissima* (syn. *Dizygotheca elegantissima* and *Schefflera elegantissima*); [74], various *Polyscias* spp. (*balfouriana*, *chinensis*, *fabian*, *fruticosa*, *guilfoylei*, and unknown species), and *Schefflera arboricola* [19,22,24,76]. Additionally, Tolba [76] tested the host range of *Xanthomonas* strains originally isolated from *H. actinophyllum* (*S. actinophylla*), and all eight strains were pathogenic on *S. arboricola*, *P. elegantissima* (*S. elegantissima*), and *H. helix*, but not on *F. japonica*. This finding was not supported by an earlier study [19]. These eight strains also failed to infect two araliaceous plants, *Aralia nudicaulis* and *Dendropanax trifidus*. The strain originally isolated from *H. canariensis* did not infect *D. trifidus* and *Tetrapanax papyriferum* [67]. Thus, the taxonomy of these pathogens remains uncertain.

Notably, Norman et al. [22] described the heterogeneity of xanthomonads isolated from various genera in the Araliaceae family, including *Hedera*, *Brassaia* (syn. *Heptapleurum*), *Polyscias*, and *Schefflera*. Based on metabolic fingerprints, fatty acid methyl ester profiles, and restriction fragment length polymorphism (RFLP) analyses, dendrogram clustering suggested that these strains should be divided into two groups: one primarily comprising *Hedera* strains and the majority of the others as *Polyscias* strains. Although advanced genotypic analyses have been recommended to verify the phylogeny and taxonomy of these xanthomonads, strains isolated from Araliaceae hosts other than ivy have not been comprehensively examined using DNA homology or genomic comparison analyses since 1984 [22,76]. Therefore, *X. hortorum* pv. *hederae* has been identified as the sole causal agent of bacterial leaf spot and leaf blight affecting six genera in the Araliaceae family: *Hedera*, *Heptapleurum*, *Fatsia*, *Plerandra*, *Polyscias*, and *Schefflera*.

**Figure 4:**
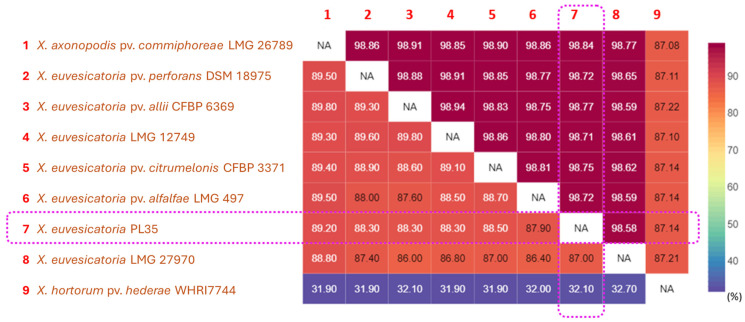
Pairwise comparison heatmap of ANI and dDDH among whole genome sequences of the panax strain PL35, the type strain of *X. hortorum* pv. *hederae*, and other related pathovars within *X. euvesicatoria* phylogenetic clade. Pairwise ANI values were calculated using CLC Genomics Workbench 22.0.2 (CLC Bio-Qiagen, Arahus, Denmark), while pairwise dDDH values were inferred using the Genome-Genome Distance Calculator (GGDC) v3.0 on the Type Strain Genome Server (TYGS) web server (https://tygs.dsmz.de/, accessed on 22 June 2025) [77,78]. Heatmaps of ANI (%) and dDDH (%) were visualized using displayR (https://www.displayr.com/, accessed on 22 June 2025). The upper triangular portion of the matrix displays ANI values, whereas the lower triangular portion displays dDDH values. The values in the matrix and heatmap bar are shown as percentages (%). The values inside the purple frames indicate that the panax strain PL35 belongs to *X. euvesicatoria*, based on the species boundary thresholds of 95% for ANI and 70% for DDH.

Chuang et al. [56,57] proposed that panax strains PL35 and A1891, isolated from *P. guilfoylei* in Hawaii, are distinct from *X. hortorum* phylogenetic clade and belong to a *X. euvesicatoria* phylogenetic clade, as determined by MLSA concatenating five housekeeping genes (Figure 3). Moreover, the Genome BLAST Distance Phylogeny (GBDP) approach and genomic relatedness values (>98% ANI and >86.7% dDDH) (Figure 4) indicated PL35 should be reclassified as *X. euvesicatoria* [56]. Additionally, Wang et al. [59] classified strains isolated from *P. guilfoylei* in Taiwan as *X. euvesicatoria* based on MLSA. They suggested assigning a novel pathovar, polysciadis, based on host specificity to *P. guilfoylei* and potentially other Araliaceae members, rather than to *Solanum lycopersicum*, *Euphorbia milii*, or *Ixora* × *westii* from other families.

## 3. Virulence Mechanisms

**Figure 5:**
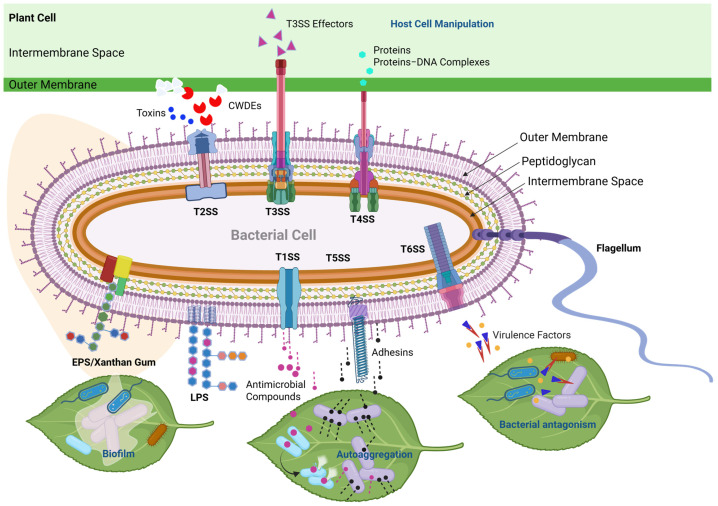
Schematic representation of type secretion systems (TSSs) and other virulence factors in *Xanthomonas* species. The T1SS serves primarily to transport various antimicrobial compounds directly from the cytoplasm to the extracellular environment [83,99]. The T2SS is a specialized secretory pathway used to export plant-cell-wall-degrading enzymes (CWDEs) as well as toxins into the extracellular space [100]. The T3SS bridges the inner and outer membranes in order to inject or translocate T3SS effector proteins directly into plant and animal cells [83,101,102,103]. The T4SS is used for bacterial conjugation and the transfer of proteins/effectors, DNA, or protein–DNA complexes from bacteria to prokaryotic or eukaryotic target cells [81,104]. The T5SS is primarily involved in exporting proteins, such as adhesins, which is crucial for host colonization and the establishment of infections in plant tissues [105]. The T6SS has been associated with contact-dependent interbacterial antagonism in *Xanthomonas* [106,107]. Moreover, the versatile T6SS also secretes virulence factors in eukaryotic hosts [108,109]. EPS/Xanthan Gum is important for the biofilm matrix, enabling the pathogen to tolerate environmental stress and protect against other microbes [84]. LPS protects bacteria from many toxic compounds and is recognized as pathogen-associated molecular patterns (PAMPs) during interactions with plants [80,94].

Successful infection of a plant by xanthomonads involves the processes of attachment, penetration, and multiplication within the plant; therefore, genes encoding exopolysaccharides (EPS), lipopolysaccharides (LPS), pili, flagella, cell-wall-degrading enzymes (CWDE), and type secretion systems (TSSs) are critical for virulence and pathogenicity [79,80,81,82,83]. The schematic representation of TSSs in xanthomonads is shown in Figure 5. The genetic makeup of pathogenicity determinants in the whole genomes of xanthomonads isolated from the Araceae and Araliaceae families is presented in Table 1. Studies on the pathogenetic mechanisms of *Xanthomonas*-associated Araceae and Araliaceae remain limited. Therefore, we summarized previous studies on how genes encoding pathogenicity and/or virulence factors influence the interactions between *Xanthomonas* spp. and their host plants. We did not include the T1SS and T5SS in this review due to the unavailability of recent genomic studies on xanthomonads associated with Araceae and Araliaceae.

### 3.1. Exopolysaccharides (EPS) and Lipopolysaccharides (LPS)

A defining characteristic of xanthomonads is the production of xanthan, an EPS with pentasaccharide-repeating units which forms a biofilm matrix enabling the pathogen to tolerate environmental stress [84]. A cluster of 12 gum genes, from *gumB* to *gumM*, plays an essential role in the assembly of lipid-linked intermediates, polymerization, and secretion of xanthan [85,86]. The gum genetic loci, spanning approximately 15 kb, are highly conserved and exhibit synteny in almost all *Xanthomonas* species. This includes Araceae strains such as *X. citri* pv. *aracearum*, *X. euvesicatoria*, *X. phaseoli* pv. *dieffenbachiae*, and *X. phaseoli* pv. *syngonii* [63]. The exceptions are *X. albilineans* and *X. theicola*, which lack the entire gum gene cluster [87,88,89,90], while the *gumN*, *gumO*, and *gumP* genes are absent in *X. fragariae* [91]. The *gumD* gene, along with *gumM*, *gumH*, *gumK*, and *gumI*, encodes glycosyltransferases that synthesize a lipid-linked pentasaccharide and is involved in the virulence of *X. campestris* pv. *campestris* on broccoli and cabbage [86,92] as well as virulence of *X. oryzae* pv. *oryzae* on rice [93]. The *gumB*, *gumC*, *gumE*, and *gumJ* genes are responsible for xanthan formation and translocation. The defective mutants of these four genes, along with *gumM* in *X. campestris* pv. *campestris*, potentially resulted in cell lethality due to the accumulation of toxic lipid-linked intermediates [86]. Additionally, knockout mutants of *gumE*, *gumI*, and *gumJ* were lethal in *X. hortorum* pv. *vitians* [89], and *gumJ* was also crucial for the survival of *X. oryzae* pv. *oryzae* [93].

LPS is a crucial component of the outer membrane (OM) of Gram-negative bacteria, consisting of a hydrophobic lipid A, a hydrophilic core oligosaccharide, and an O-antigen (OA) polysaccharide chain. It protects bacteria from many toxic compounds and is recognized as pathogen-associated molecular patterns (PAMPs) during interactions with plants and animals [80,94]. The LPS gene cluster, i.e., the wxc gene cluster, is located between the highly conserved metB and etfA genes, which encode for cystathionine gamma-synthase and the electron transport flavoprotein subunit alpha proteins, respectively [95,96]. Situated between *etfA* and *metB*, the number of genes and their sequence similarity exhibit significant variability across *Xanthomonas* genomes, with the exception of two conserved genes, *wzm* and *wzt*. These genes are involved in the ATP-binding cassette (ABC) transporter pathway [97,98].

In aroid strains, variability in LPS was observed, ranging from the shortest length of 19.7 kb in *X. citri* pv. *aracearum* LMG 7399 to the longest length of 25.9 kb in *X. phaseoli* pv. *dieffenbachiae* LMG 25940 [63]. Both the wzm gene, encoding the O-antigen-permease protein, and the *wzt* gene, encoding the teichoic ATP-binding protein, were present in *X. phaseoli* pv. *dieffenbachiae* LMG 25940, *X. phaseoli* pv. *syngonii* LMG 9055, and *X. citri* pv. *aracearum* LMG7399. However, only the gene encoding the teichoic ATP-binding protein was found in *X. euvesicatoria* LMG 12749 [63]. When comparing the type strain *X. hortorum* pv. *hederae* CFBP 4925T isolated from ivy (Araliaceae) with other pathovars, *X. hortorum* pv. *cynarae* CFBP 4188PT (formerly named *X. cynarae*) from artichoke and *X. hortorum* pv. *gardneri* ATCC 19865PT (formerly named *X. gardneri*) from tomato, *wzm* and *wzt* homologs were identified in all three genomes. Average nucleotide differences of 69.5 in the *metB* genes and 24 in the *etfA* genes were observed [110].

The OA precursor gmd (GDP-mannose 4,6-dehydratase encoding gene) and rmd (GDP-4-dehydro-D-rhamnose reductase encoding gene) were reported in *X. axonopodis* pv. *malvacearum*, *X. campestris* pv. *campestris* ATCC 33913, *X. campestris* pv. *armoraciae* 756C, *X. euvesicatoria* 85–10, *X. fuscans* subsp. *fuscans* 4834-R, and three pathovars of *X. hortorum* (pv. *hederae* CFBP 4925T, pv. *cynarae* CFBP4188PT, and pv. *gardneri* ATCC19865PT) but not in *X. citri* pv. *citri* strain 306 or the two *X. oryzae* pathovars strains BLS 256 and MAFF 311018 [95,110,111,112]. The highly polymorphic LPS loci were attributed to multiple horizontal gene transfer (HGT) and recombination events, especially because several insertion sequence (IS) elements were found in *X. oryzae* pv. *oryzicola* BLS 256, *X. oryzae* pv. *oryzae* MAFF 311018, and *X. campestris* pv. *campestris* ATCC 33913 and 8004 [111,113].

In *X. citri* pv. *citri*, which causes citrus canker disease, gene disruption mutants of *wxacO* (encoding a putative transmembrane protein), *rfbC* (encoding a truncated O-antigen biosynthesis protein), and *nlxA* (encoding a putative glycosyltransferase) within the LPS cluster were found to be more sensitive to the antimicrobial peptide polymyxin B, hydrogen peroxide, and other environmental stresses. These mutants also showed impaired swimming and swarming motility and exhibited reduced virulence on grapefruit leaves when spray inoculated [114,115]. Interestingly, mutations in the LPS biosynthesis genes influenced EPS production, as LPS and EPS share some common genetic determinants including precursor molecules at the level of nucleotide sugar metabolism in polysaccharide biosynthesis [95,115,116]. The yield of xanthan produced by the deficient mutants of the *wxcB*, *wxcK*, and *wxcN* genes in *X. campestris* pv. *campestris* increased, while the yield of EPS produced by the *nlxA* mutant of *X. citri* pv. *citri* decreased [115,116].

### 3.2. Type II Secretion System (T2SS)

The Type II secretion system (T2SS) is a highly specialized secretory pathway utilized by Gram-negative bacteria to export plant-cell-wall-degrading enzymes (CWDEs) such as cellulases, pectinases, and xylanases, along with toxins, lipases, proteases, and phospholipases into the extracellular space [100]. The T2SS is comprised of two distinct gene clusters, *xcs* and *xps*, which are conserved within the proteobacterial family, including *Xanthomonas*. However, only the intact *xps* cluster is present in *X. albilineans*, *X. hyacinthi*, *X. sacchari*, *X. theicola*, *X. fragariae*, *X. populi*, *X. bromi*, *X. axonopodis* pv. *vasculorum*, *X. vasicola* pathovars *vasculorum* and *musacearum* (syn. *X. axonopodis* pv. *musacearum*), and *X. oryzae* pathovars *oryzae* and *oryzicola* [83,111,117,118]. The xcs gene cluster contains 12 genes, including the *xcsC* gene encoding a PDZ-domain-containing protein and genes from *xscD*/*gspD* to *xscN*/*gspN*. On the other hand, the *xps* gene cluster is comprised of 11 genes, including *xpsE*/*gspE* to *xpsK*/*gspK*, the *xpsL* gene encoding a *pilN*-domain-containing protein, and genes from *xpsM*/*gspM* to *xpsN*/*gspN*, along with *xpsD*/*gspD*. Both the *xcs* and *xps* gene clusters are highly conserved among xanthomonads isolated from the Araceae and Araliaceae families, including *X. citri* pv. *aracearum*, *X. euvesicatoria*, *X. phaseoli* pv. *dieffenbachiae*, *X. phaseoli* pv. *syngonii*, and *X. hortorum* pv. *hederae* [63,118].

The virulence function of *xps* gene clusters has appeared in several xanthomonads. However, there have been no reports demonstrating the contribution of *xcs* genes to virulence, except for a study by Szczesny et al. [119], which suggested that *xcs* genes could partially complement the homologous *xpsE* and *xpsD* genes [83]. Hu et al. [120] investigated ten Xps pleiotropic mutants of *X. campestris* pv. *campestris*, which were non-pathogenic to cabbage and exhibited reduced accumulation of amylase, endoglucanase, and polygalacturonate lyase. Baptista et al. [121] demonstrated that the absence of the *xpsD* gene in *X. axonopodis* pv. *citri* (syn. *X. citri* pv. *citri*), which is crucial for the secretion of various enzymes including cellulases, resulted in the reduced virulence of the pathogen on citrus. Similarly, reduced symptoms on rice were observed in *xpsF* and *xpsD* mutants of *X. oryzae* pv. *oryzae* due to an export deficiency of xylanase [122,123]. In *X. campestris* pv. *vesicatoria* 85-10 (heterotypic syn. *X. euvesicatoria*), the *xpsD* gene was found to contribute significantly to virulence, unlike the *xcsD* genes, while the *xpsE* gene influenced T3SS-dependent effector protein translocation [119]. Furthermore, defective mutants of T2SS secreted substrates, such as xylanase and protease, which were reported to affect the virulence of *X. campestris* pv. *vesicatoria* in tomatoes [124].

### 3.3. Type III Secretion System (T3SS)

The Type III secretion system (T3SS), consisting of more than 20 proteins encoded by *hrp* (hypersensitive response and pathogenicity) and *hrc* (hrp conserved) genes, is a contact-dependent pathway. It forms an apparatus located between the inner and outer membranes to inject or translocate proteins into plant and animal cells [83,101,102,103]. Most T3SS membrane-associated proteins are conserved among different pathogenic bacteria [125], and nine of these proteins, including HrcC, HrcJ, HrcN, and HrcQ to HrcV, are highly conserved hrc-gene-encoded proteins in plant and animal pathogens [126]. Interestingly, genes encoding components of the Type III secretion system (T3SS) share high homology with clustered genes involved in flagellar biosynthesis [103]. Among the Xanthomonas species, the manipulation of pathogenicity by the protein repertoire, termed type III effectors (T3Es,) secreted via T3SS [127], has been studied for more than two decades. White et al. [128] categorized T3Es into 39 classes based on sequence relatedness, identifying various biochemical or structural motifs, including glycerolphosphoryl diester phosphodiesterase, site-specific DNA binding, nuclear localization, M27 zinc protease, and tyrosine phosphatase, among others. A comparison of the genomes from seven *Xanthomonas* species revealed that eleven T3E groups, such as avirulence Bs2 (AvrBs2), *Xanthomonas* outer protein F (XopF), XopK-N, XopP-R, XopX, and XopZ, were commonly present. In contrast, XopB, XopD, XopT-U, XopW, XopAC, XopAF, XopAH, XopAJ, and XopAK showed restricted distribution across the seven genomes [128].

The Type III secretion system (T3SS) has been confirmed to play a crucial role as a pathogenicity determinant, with extensive research focused on gene function. Interestingly, it has been reported that some non-pathogenic *Xanthomonas* strains lack the Hrp T3SS and its associated substrates. These include *X. sacchari* NCPPB 4393 and R1 strains [64,65], the *X. maliensis* type strain CFBP 7942 [129], the sugarcane-pathogenic *X. albilineans* GPE PC73 strain [130], and the cannabis-infecting *X. cannabis* pv. *cannabis* strains NCPPB 2877 and NCPPB 3753 [131]. Merda et al. [132] analyzed the T3SS gene cluster and its upstream and downstream genomic contexts (within a 20 kb window) across 82 genomes of various *Xanthomonas* species/pathovars. They discovered similar *hrp* gene clusters in the genome of *X. hortorum* pv. *hederae*, comprising 20 genes isolated from the Araliaceae family, and in the genomes of *X. arboricola* pv. *zantedeschiae* (24 genes) and *X. phaseoli* pv. *dieffenbachiae* (22 genes), both isolated from the Araceae family. Genomic rearrangement events were observed on both sides of the 20 kb window in *X. arboricola* pv. *zantedeschiae* and *X. phaseoli* pv. *dieffenbachiae*. However, only one side of the 20 kb flanking region was analyzed in *X. hortorum* pv. *hederae*, which was presumed to be one of the main gene donors in the Maximum Likelihood (ML) phylogeny [132].

Almost all syntenic T3SS, located between the XopAE- and Hpa2-encoding genes, was displayed in four aroid strains. The core *hrp* gene cluster consisted of 27–30 genes encoding *Hrp* components, Xop effectors, and hypothetical proteins [63]. Notably, additional mobile elements located in the vicinity of the hrp cluster were observed in the *X. euvesicatoria* LMG 12749 strain from *Philodendron* but were absent in the other three aroid strains (LMG 25940 from *Anthurium*, LMG 7399 from *Dieffenbachia*, and LMG 9055 from *Syngonium*) [63]. Similar to *X. campestris* pv. *vesicatoria* 85-10, *X. oryzae* pv. *oryzae* PXO86, and *X. fuscans* subsp. *fuscans* 4834-R strains, mobile genetic elements flanking both sides of the hrp gene cluster were proposed to carry virulence genes and act as a pathogenicity island [112,133]. The highest number of T3Es was predicted in *X. euvesicatoria* LMG 12749 among the four aroid strains; however, the LMG 12749 strain was very weakly pathogenic to the tested aroids. In contrast, *X. citri* pv. *aracearum* LMG 7399 with 24 T3Es, *X. phaseoli* pv. *syngonii* LMG 9055 with 22 T3Es, and *X. phaseoli* pv. *dieffenbachiae* LMG 25940 with 20 T3Es caused disease on the tested aroids and shared some T3Es (XopE2, XopG, and XopAM) that were lacking in the LMG 12749 strain [63].

From previous studies, the *hrp*, *hrc*, *hpa* (*hrp*-associated), and effector genes involved in the T3SS have shown various effects on pathogenicity across different *Xanthomonas*–host pathosystems [134]. For the *X. campestris* pv. *vesicatoria* strain 85-10, which contains the avrBs1 avirulence gene, deletion mutants of hrpB1, hrpB2, hrpB4, and hrpB5 in the hrpB operon were unable to infect susceptible pepper plants or to induce a hypersensitive response (HR) in resistant pepper plants [134,135]. With the exception of the *hrpF* gene, mutants deficient in *hrp-hrc* genes (including *hrpE*, *hrpD6*, *hrpD5*, *hrcS*, *hrcR*, *hrcQ*, *hrcV*, *hrcU*, *hrpB1*, *hrpB2*, *hrcJ*, *hrpB4*, *hrpB5*, *hrcN*, *hrpB7*, *hrcT*, *hrcC*, *hrpG*, and *hrpX*) derived from *X. oryzae* pv. *oryzae* KACC10859 were unable to cause disease in rice. By contrast, the *hrpF* null mutant of *X. campestris* pv. *vesicatoria* demonstrated that HrpF is required for pathogenicity to pepper and acts as a translocon protein at the plant–pathogen interface [135]. Similar results were observed in the *X. campestris* pv. *vesicatoria* and soybean pathosystem [136]. Additionally, the *hpaA*, *hpaF*, *hpaP*, *hpa1*, and *hpa2* disruption mutants of *X. oryzae* pv. *oryzae* KACC 10859 exhibited various levels of defective virulence, with the exception of the hpaB disruption mutant, which completely lost its pathogenicity.

### 3.4. Type IV Secretion System (T4SS)

The Type IV secretion system (T4SS) is a highly complex and versatile multiprotein system for bacterial conjugation and transferring proteins/effectors, DNA, and/or protein–DNA complexes from bacteria to prokaryotic or eukaryotic target cells [81,104]. Three types of T4SS loci are found on the chromosomes and/or plasmids of Gram-negative bacteria, such as the virB, trb, and avhB loci in *Agrobacterium tumefaciens*. In *X. citri* pv. *citri*, there are chromosomal *virB* loci and plasmid-borne *twr/virB* loci, while *X. campestris* pv. *campestris* possesses only a single virB cluster [113,137,138]. The canonical T4SS was assembled by 12 core proteins including the periplasmic transglycosylase VirB1, a periplasmic VirB7–VirB9–VirB10 repeated core complex, an inner membrane VirB3–VirB6–VirB8 complex, the outer membrane lipoprotein VirB7, VirB2–VirB5-formed extracellular pilus, and three membrane-associated ATPase (VirB4 and VirB11 plus VirD4) [139,140,141]. The ATPase VirD4 is capable of recognizing and interacting with proteins that have C-terminal XVIPCDs (Xanthomonas-VirD4-interacting protein conserved domains) and/or protein–DNA complexes [138]. Furthermore, these toxic XVIPs are secreted by the T4SS to kill other bacterial cells. For instance, virB7 and *virD4* gene-defective mutants of *X. citri* pv. *citri* lost the ability to lyse *Escherichia coli* while being co-cultured [141].

In the study by Constantin et al. [63], a *virB5*-like gene was identified in aroid strains, exhibiting approximately 35% similarity with X. euvesicatoria 85-10. All core protein-encoding genes, with the exception of *virB5*, were found in *X. euvesicatoria* LMG 12749 and *X. phaseoli* pv. *dieffenbachiae* LMG 25940, sharing 68 to 96% sequence homology. In contrast, only *virB10* and *virB11* were present in *X. phaseoli* pv. *syngonii* LMG 9055, while virB6 and a truncated virD4 were harbored in *X. citri* pv. *aracearum* LMG 7399 [63]. Similar to *X. hortorum* pv. *hederae*, the *virB3-B4*, *virB8-virB11*, and *virD4* genes were found in pathovars *vitians*, *pelargonii*, and *taraxaci* [142]. According to subsequent studies, the T4SS of *Xanthomonas* spp. was shown to indirectly affect pathogenicity on their hosts [143]. The *virB7* knockout mutant of *X. citri* pv. *citri* developed symptoms similar to the wild type on citrus leaves [144], and a 10-gene deletion mutant of *X. campestris* pv. campestris, ranging from *virD4* to *virB4*, exhibited unchanged virulence on tested cabbage, Chinese cabbage, Chinese kale, pak choi, and radish [145].

### 3.5. Type VI Secretion System (T6SS)

In most Gram-negative bacteria, the Type VI secretion system (T6SS) serves as another contact-dependent secretion machinery that translocates virulence factors, including effectors and toxins, into both prokaryotic and eukaryotic cells [108,109]. This contractile injection system consists of three main components: (i) the membrane-spanning complex, formed by proteins encoded by *tssJ*, *tssL*, and *tssM* and (ii) a baseplate, assembled by proteins encoded by *tssE-G*, *tssK*, and *tssI/vgrG* along with PAAR repeats, with the formation of (iii) an extended inner tube facilitated by the Hcp (Haemolysin-Coregulated Protein) encoded by the *tssD/hcp* gene. This tube is assembled by proteins encoded by *tssB* and *tssC* and is coordinated by a TssA-like protein [83,146,147]. Three different subclasses of T6SS loci, comprising 13 core genes, have been reported in *Xanthomonas* spp. However, none of these were found in *X. campestris*, *X. hyacinthi*, *X. theicola*, *X. sacchari*, and *X. albilineans* [83,118,148].

Moreover, the T6SS subclasses (T6SS-I, II, and III) acquired via horizontal gene transfer events exhibited various organization and synteny of core genes among Xanthomonas spp. [149]. Both T6SS-I/T6SS-3 and T6SS-III/T6SS-3 were present *in X. phaseoli* pv. *dieffenbachiae* LMG 25940 and *X. phaseoli* pv. *syngonii* LMG 9055, as well as in *X. euvesicatoria* strains including LMG 12749 from aroids, 85-10 from pepper, 91-118 and CFBP 7293 from tomato, and CFBP 3836 (syn. *X. alfalfae* pv. *alfalfae*) from alfalfa, and F1 (syn. *X. axonopodis* pv. *citrumelonis*) from citrus [63,118,150]. The *Xanthomonas citri* pv. *aracearum* LMG 7399 strain possessed only the T6SS-III/T6SS-3 subclass, closely clustered with the T6SS-III of *X. phaseoli* pv. *dieffenbachiae* LMG 25940 and *X. phaseoli* pv. *syngonii* LMG 9055, while *X. hortorum* pv. *hederae* WHRI 7744 from ivy (Araliaceae) contained only the T6SS-II/T6SS-4 subclass, also present in *X. oryzae* pathovars and *X. fragariae* [18,63,118,150].

Besides its role in antagonistic activity within the bacterial community, the versatile T6SS has been involved in biofilm formation, antibacterial activity, and virulence in eukaryotic hosts [106,107,151]. The T6SS secretion system is crucial for *X. citri* pv. *citri* to defend against soil amoebae, which are bacterial predators that feed in a phagocytic manner [152]. Montenegro Benavides et al. [149] revealed that the *vgrG*, *hcp*, and *clpV* genes of *X. phaseoli* pv. *manihotis*, possessing only T6SS-III, were essential for maximizing aggressiveness on cassava, while the *clpV* gene also significantly impaired motility. The function of the *hcp* gene in T6SS-I, but not in T6SS-II of *X. oryzae* pv. *oryzae*, was demonstrated to play a role in virulence to rice [153]. In contrast, neither the hcp gene in T6SS-I nor the *hcp* gene in T6SS-II of *X. oryzae* pv. *oryzicola* impacted virulence on rice [154]. Furthermore, the *tssM* mutant in T6SS-III of *X. perforans* (syn. *X. euvesicatoria* pv. *perforans*) [13,14] exhibited increased virulence on tomato leaves during dip inoculation and no significant difference in severity during infiltration infection [155]. Notably, two *tssM* genes, sharing 65% identity in two T6SS loci (I and III), were found in *X. euvesicatoria* pv. *perforans*, and the one in T6SS-III was required for successful epiphytic survival during the asymptomatic phase of tomato infection [155]. Gene expression profiles of *X. citri* pv. *citri*, including *tssB*, *clpV*, *vgrG*, and *ecfK* (a T6SS regulator-encoded gene), were increased during epiphytic growth rather than inside the mesophyll of sweet orange [156].

### 3.6. Cell-Wall-Degrading Enzymes (CWDEs)

*Xanthomonas* spp. secrete a diverse array of cellulolytic, hemicellulolytic, and pectinolytic enzymes, collectively termed cell-wall-degrading enzymes (CWDEs), which degrade the plant cell wall matrix. An average of 31 homologs of the CWDE repertoire was retrieved from 26 xanthomonad genomes including complex groups of cellulases, cellobiosidases, polygalacturonase, pectin methylesterases, pectate lyases, rhamnogalacturonases, rhamnogalacturonan acetylesterase, xylanase, and beta-glucosidases xylosidases/arabinosidases [91]. Vieira et al. [157] unraveled a xyloglucan utilization locus comprised of two TonB-dependent transporters, four glycoside hydrolases, and one esterase, which is highly conserved in 34 *Xanthomonas* species/pathovars that infect monocots and dicots (excluding *X. oryzae*, *X. translucens*, and *X. albilineans*). Previous studies have reported that xanthomonads mainly manipulated the T2SS to secrete CWDEs into extracellular milieu, including the following: xylanases (XCV0965, XCV4358, and XCV4360), protease (XCV3671), and lipase (XCV0536) as T2SS-dependent substrates in *X. campestris* pv. *vesicatoria* 85-10 [119,124], cellobiosidase (CbsA), cellulose (ClsA), and lipase/esterase (LipA) via T2SS in *X. oryzae* pv. *oryzae* [158,159]. Moreover, an alternative transport route for some T2SS substrates was proposed to attribute to the CWDEs presenting in outer membrane vesicles (OMVs), also called type zero secretion system (T0SS). T2SS-secreted cellulose (Egl) and xylosidase (XynB) were observed in OMVs of X. campestris pv. campestris [160]. Moreover, putative protease (XCV0007), xylanase (XCV4355), and other T2S substrates including lipase (XCV0536), protease (XCV3671), and xylanases (XCV4358 and XCV4360) were detected in OMVs of *X. campestris* pv. *vesicatoria* under immune-electron microscopy [124]. Based on a previous genomic study of aroid strains isolated from *dieffenbachia*, *anthurium*, *philodendron*, and *syngonium*, the two strains possessing the highest number of CWDEs are *X. phaseoli* pv. *dieffenbachiae* LMG 25940 (total = 40) with the most pectinolytic and hemicellulolytic enzymes and *X. citri* pv. *aracearum* LMG7399 (total = 38) having the most cellulolytic enzymes [63]. Meanwhile, *X. euvesicatoria* LMG12749 contained the identical number of hemicellulolytic enzymes as LMG 25940 but fewer than the other two groups. Although the *X. phaseoli* pv. *syngonii* LMG 9055 strain was the most aggressive on *syngonium*, it displayed the lowest combination of CWDEs due to truncated or frameshifted sequences and incomplete genome-assembly (232 contigs, N50: 52,766 bp) [63]. The large CWDE repertoire was reported in the genomes of *X. hortorum* pv. *vitians* LM 16734 isolated from lettuce and pv. gardneri ATCC 19865 from tomato; however, no details were discussed for *X. hortorum* pv. *hederae* from ivy [89,91].

In *X. campestris* pv. *vesicatoria* strain 85-10, xylanase (XynC, XCV0965) contributed to bacterial virulence on pepper, and the *xynC*-deficient mutant showed reduced leaf spot symptoms and lower in planta bacterial multiplication [119]. Two Xyn homologs, XynA and XynB, were found in *X. oryzae* pv. *oryzae*, though only XynB displayed dominantly xylanase activity [158]. The pathogenicity of the *xynB* gene and *lipA-xynB* double mutants of *X. oryzae* pv. *oryzae* was reduced significantly [158]. Moreover, the *cbsA* disruption mutant and *lipA clsA* double mutant abolished the virulence of *X. oryzae* pv. *oryzae* to rice, whereas *clsA*- and *lipA*-deficient mutants partially reduced virulence [158,159]. In comparison with cellulase-, xylanase-, and lipase-encoding genes, protease- and pectin-homogalacturonan (HG)-degrading genes of *X. oryzae* pv. *oryzae* showed inefficient virulence to rice. Tayi et al. [161] reported four HG degrading genes including *pglA* encoding polygalacturonase, pmt encoding pectin methyl esterase, and both *pel* and *pelL* encoding pectate lyases, which played minor roles in virulence to rice. Interestingly, *ecpA* encoding extracellular protease was reported not to affect virulence on rice for *X. oryzae* pv. *oryzae*, but the gene is a crucial virulence determinant required for *X. oryzae* pv. *oryzicola* to infect rice [162]. Besides CWDEs, non-fimbrial adhesion-like protein A (XadA) promoted the virulence of *X. oryzae* pv. *oryzae* to rice [163].

### 3.7. Flagellum

The flagellum is a complex motility organelle that enables bacteria to swim and swarm. Besides motility, it facilitates adherence to hosts, biofilm formation, protein export, and cellular invasion [164,165,166]. A typical flagellum consists of a cytoplasmic export apparatus, body rings including L, P, MS, and C rings embedded in the cell membrane, tubular axial structures including a hook and rod across the cell membrane, and filaments outside of the membrane [167]. The flagellar structures, composed of ~20,000–30,000 protein subunits of over 30 different proteins, are assembled in a sequential order: basal rings, export apparatus, rod, hook, and then filaments [167,168]. For xanthomonads having a single polar flagellum, four gene clusters are involved in flagellar synthesis and regulation: cluster I contains *fliSDC*, *flgLKJIHGFEDCB*, *cheV*, and *flgAM* genes. Cluster II consists of *fliA*, *fleN*, *flhFAB*, and *fliRQPONMLKJIHGFE* genes; cluster III possesses only two flagellar motor protein encoding genes, *motA* and *motB;* cluster IV contains chemotaxis protein, methyl-accepting chemotaxis protein, partitioning protein, and motor protein genes [63,82]. The flagellar gene clusters I, III, and IV were conserved in *X. citri* pv. *aracearum*, *X. euvesicatoria*, *X. phaseoli* pv. *dieffenbachiae*, and pv. *syngonii* strains isolated from Araceae, but cluster II showed differences in the gene organization and the number of hypothetical protein-coding genes [63]. No information about flagellar biosynthesis gene clusters in *X. hortorum* pv. *hederae* is presently available.

Mutations in either flagellar component genes or regulatory genes of different xanthomonads affect diverse flagellation, motility, and pathogenicity on their hosts. In the study by Darrasse et al. [112] the integrity of the flagellar gene cluster was examined across various *Xanthomonas* species and pathovars. Among the 38 strained tested, 4.4% including *X. albilineans* CFBP 2523, *X. arboricola* pv. *corylina* CFBP1159, *X. cucurbitae* CFBP2542, *X. fuscans* subsp. *fuscans* CFBP 4885, and *X. sacchari* CFBP 4641 were non-motile, due to deficiencies in their flagellar gene clusters. Interestingly, the non-flagellated *X. fuscans* subsp. *fuscans* CFBP 4885 is highly pathogenic on beans [112]. In *X. campestris* pv. *campestris*, the insertion mutants of the flhA gene, which encoded a subunit of an export apparatus, and of the *fliA* gene, which encoded sigma factor 28 in cluster II, were defective in flagellation and motility, and both mutations caused attenuated virulence on a cabbage leaf [169]. Conversely, *flhB-* and *fleN*-deficient mutants of *X. campestris* pv. *campestris* displayed identical virulence to parental strains, even though a *flhB*-deficient mutant was immotile and non-flagellated [169], but a *fleN*-deficient mutant was hyper-flagellated [170]. *Xanthomonas oryzae* pv. *oryzae* mutants with a mutation in the *flhF* gene, flanking in the downstream of *flhB* and *flhA*, showed no interference in flagellation, motility, and pathogenicity to rice using scissor-dipping and spray methods [171]. Whereas the *flhF* deletion mutant of *X. campestris* pv. *campestris* produced one to two lateral flagella like *fliA* and *flgM* mutants, the *flhF* mutant was diminished in pathogenicity to cabbage [172].

Two *rpoN* homologous regulators, RpoN1 and RpoN2, were revealed in *X. citri* subsp. *citri* [173] and *X. campestris* pv. *campestris* [169]. Gene *rpoN2* is in a large flagellar gene cluster I while gene *rpoN1* is harbored in a phosphotransferase system [169,173,174]. *Xanthomonas citri* subsp. *citri rpoN1* and *rpoN2* deletion mutants and double mutants were delayed in canker symptom development; however, the distinct regulatory effects on motility were observed in these mutants [173]. Yang et al. [169] and Li et al. [174] reported the mutation of *rpoN2* in *X. campestris* pv. *campestris* strains ATCC33913 and Xc1, respectively, not only reduced flagellation and motility but also significantly affected the virulence to susceptible cabbage. The direct regulation of RpoN2 with corresponding promoters of genes *fliC* and *fliQ* was revealed while analyzing transcriptomic gene expression profiles [174], whereas a knockout mutant of the *rpoN1* gene, harbored in a phosphotransferase system, caused similar symptoms to the Xc1 strain. Also, a *rpoN1N2* double mutant lost pathogenicity and motility, similar to a *rpoN2* gene knockout mutant, and plasmid-born *rpoN2* complemented the defective double mutant, but plasmid-born *rpoN1* failed to restore swimming ability [174]. Taken together, homologous regulator RpoN2 worked independently and was not interchangeable with RpoN1 [173,174].

## 4. Conclusions and Future Directions

In conclusion, recent advances in phylogenomic and pathogenicity studies have led to the reclassification of *Xanthomonas* strains from Araceae into six species (*X. arboricola*, *X. citri*, *X. euvesicatoria*, *X. hawaiiensis*, *X. phaseoli*, and *X. sacchari*) and those from Araliaceae into two species (*X. hederae* and *X. euvesicatoria*). Pathogenicity and host range tests identified several pathovars among strains from both the Araceae and Araliaceae families, including *X. arboricola* pv. *zantedeschiae*, *X. citri* pv. *aracearum*, *X. euvesicatoria* pv. *amorphophalli*, *X. euvesicatoria* pv. *polysciadis*, *X. hortorum* pv. *hederae*, *X. phaseoli* pv. *dieffenbachiae*, and *X. phaseoli* pv. *syngonii* (Figure 2). This refined taxonomy provides a more robust foundation for disease diagnostics, surveillance, and management in economically important ornamental crops. However, the phylogenetic placement of certain strains from diverse araceous and araliaceous hosts remains unresolved due to limited genomic representation. To address these gaps, future research should prioritize comprehensive genome sequencing of underrepresented strains, especially from genera such as Epipremnum and Rhaphidophora, and apply comparative pan-genome and population genomic analyses to clarify species boundaries and pathovar affiliations. Additionally, functional genomics and reverse genetic studies targeting key pathogenicity determinants—such as secretion systems, exopolysaccharide and lipopolysaccharide biosynthesis, and cell-wall-degrading enzymes—will be essential to unravel the molecular mechanisms underpinning host adaptation and virulence. By integrating high-throughput sequencing with targeted functional assays, the field is poised to resolve outstanding taxonomic ambiguities and accelerate the development of precise diagnostic tools and effective disease management strategies, thereby advancing both the fundamental understanding and practical control of *Xanthomonas* diseases in Araceae and Araliaceae.

## Figures and Tables

**Table 1:** Overview of pathogenicity-related gene clusters in *Xanthomonas* species/pathovars isolated from Araceae and Araliaceae.

Pathogenicity-Related Gene Clusters	*X. phaseoli* pv. *dieffenbachiae* (*Anthurium*)	*X. phaseoli* pv. *syngonii* (*Syngonium*)	*X. euvesicatoria* (*Philodendron*)	*X. citri* pv. *aracearum* (*Dieffenbachia*)	*X*. *hortorum* pv. *hederae* (*Hedera*)
Exopolysaccharide	Yes (all 12 *gum* genes)	Yes (all 12 *gum* genes)	Yes (all 12 *gum* genes)	Yes (all 12 *gum* genes)	NA
Lipopolysaccharides	Yes (with *wzm* and *wzt* between *metB* and *etfA*)	Yes (with *wzm* and *wzt* between *metB* and *etfA*)	Yes (with only *wzt* between *metB* and *etfA*)	Yes (with *wzm* and *wzt* between *metB* and *etfA*)	Yes (with *wzm* and *wzt* between *metB* and *etfA*)
Type II secrete system Xcs	Yes (all 12 *xcs* genes)	Yes (all 12 *xcs* genes)	Yes (all 12 *xcs* genes)	Yes (all 12 *xcs* genes)	Yes (all 12 *xcs* genes)
Type II secrete system Xps	Yes (all 11 x*ps* genes)	Yes (all 11 x*ps* genes)	Yes (all 11 x*ps* genes)	Yes (all 11 x*ps* genes)	Yes (all 11 x*ps* genes)
Type III secrete system	Yes (23 *hrp*, *hrc*, and *hpa* genes)	Yes (23 *hrp*, *hrc*, and *hpa* genes)	Yes (23 *hrp*, *hrc*, and *hpa* genes)	Yes (23 *hrp*, *hrc*, and *hpa* genes)	Yes (20 *hrp*, *hrc*, and *hpa* genes)
Type II secrete effector	20	22	27	24	NA
Type IV secrete system	Yes (11 genes, no *virB5*)	Yes (2 genes, *virB10* and *virB11*)	Yes (11 *vir* genes, no *virB5*)	Yes (2 genes, *virB6* and truncated *virD4*)	Yes (7 genes, *virB3-4*, *virB7-11*, and *virD4*)
Type VI secrete system T6SS-I/T6SS-3	Yes	Yes	Yes	No	No
Type VI secrete systemT6SS-II/T6SS-4	No	No	No	No	Yes
Type VI secrete systemT6SS-III/T6SS-3	Yes	Yes	Yes	Yes	No
Cell-wall-degrading enzyme	40	27	33	38	NA
Flagellar biosynthesis	Yes (all 4 clusters)	Yes (all 4 clusters)	Yes (all 4 clusters)	Yes (all 4 clusters)	NA

NA indicates that the data is not available.

## Data Availability

Not applicable.
